# Residential proximity to industrial zones and self-reported respiratory morbidity: a community-based cross-sectional survey of three Karachi industrial areas

**DOI:** 10.3389/fpubh.2025.1672049

**Published:** 2025-11-24

**Authors:** Kiran Shahbaz, Shiraz Shaikh, Aroosa Nighat, Irfanullah Khan, Mehran Ullah, Farkhanda Shaheen

**Affiliations:** 1 Department of APPNA Institute of Public Health, Karachi, Pakistan; 2 Information Systems and Operations Management Department, King Fahd Business School, King Fahd University of Petroleum and Minerals, Dhahran, Saudi Arabia; 3 Interdisciplinary Research Centre for Smart Mobility and Logistics, King Fahd University of Petroleum and Minerals, Dhahran, Saudi Arabia; 4 School of Business and Creative Industries, University of the West of Scotland, Paisley, Scotland, United Kingdom; 5 Department of Biomedical Sciences, Faculty of Health and Biomedical Sciences, Ziauddin University, Karachi, Pakistan

**Keywords:** industrial air pollution, proximity to industrial zones, residents, respiratory health, asthma, chronic bronchitis, Karachi

## Abstract

**Background:**

Industrial emissions in Karachi contribute to poor ambient air quality and may adversely affect nearby residents’ respiratory health. This study assessed whether residential distance from industrial zones is associated with respiratory symptoms and chronic respiratory disease.

**Methods:**

We conducted a comparative, community-based cross-sectional survey (March–August 2024) of 462 adults sampled equally around three industrial zones S. I. T. E., Korangi, and Landhi (*n* = 154 each). Residential distance to the nearest industrial zone was classified as within 5 km vs. more than 5 km (distance estimated from mapped home addresses). Respiratory outcomes (symptoms; chronic bronchitis; asthma) were obtained via a validated questionnaire, and multivariable logistic regression estimated adjusted associations. *This was a cross-sectional, community-based study using self-reported data without clinical assessments.*

**Results:**

Compared with residents living more than 5 km away, those living within 5 km reported substantially higher prevalences of cough, phlegm, wheeze, and dyspnea. Living more than 5 km from an industrial zone was independently associated with markedly lower odds of chronic bronchitis (adjusted OR 0.09, 95% CI 0.02–0.72) and asthma (adjusted OR 0.14, 95% CI 0.03–0.67). Higher education was protective for both outcomes, and regular mask use was protective for chronic bronchitis; smoking and industrial employment were associated with greater respiratory morbidity.

**Conclusion:**

In Karachi, residing within 5 km of major industrial zones is linked to a higher burden of self-reported respiratory symptoms and chronic respiratory disease. These findings underscore the importance of early screening and preventive strategies for nearby communities and support zoning and urban-planning measures that increase residential buffers from industrial facilities.

## 1 Introduction

Air pollution, which can harm both humans and the environment, is caused by hazardous compounds in the Earth’s atmosphere (1, 2). There are two primary sources of air pollution: natural and man-made. Natural sources include dust storms and volcanic eruptions; man-made sources include poor waste management, vehicle emissions, industrial activities, and agricultural practices (2). Among the various air pollutants released by industries are particulate matter, nitrogen oxides, sulfur dioxide, carbon monoxide, and volatile organic compounds (3). Along with burning fossil fuels like coal, oil, and gas, industrial processes, including metal processing, paper production, and chemical manufacturing, are the main sources of air pollution produced by industry (4). Air pollution can also destroy the environment, harming crops, plantations, and waterways (5). Air pollution causes a wide range of ill health effects, both long-term and short-term, including respiratory illnesses and cancer (6). Respiratory issues may include asthma, bronchitis, and chronic obstructive pulmonary disease (COPD) (7, 8), with symptoms such as coughing, wheezing, and shortness of breath (9). Air pollution can also raise the risk of cardiovascular problems such as excessive blood pressure and artery damage leading to heart attack and stroke (10). The effects on the nervous system include causing dizziness, headaches (11). It can affect the reproductive system, too, bringing about infertility, low birth weight, and premature birth (12). Skin and eye problems also arise as a direct or indirect outcome of air pollution, including rashes and skin irritation, and eye irritation (13). According to the World Health Organization, breathing conditions account for 6.7% of all deaths globally, making it the 3rd main reason for loss of life after cardiovascular sicknesses and cancer (13). Air pollution is an important contributor to respiratory disease globally and regionally; exposure to air pollution is estimated to cause 4.2 million premature deaths yearly, with respiratory disease being one of the leading causes of this mortality (8). Nearly 90% of deaths from chronic obstructive pulmonary disease (COPD) occur in low and middle-income countries (LMICs), primarily due to industrial air pollution and other environmental factors (7). In a survey conducted by Wen-Wen Chang in Argentina (La Plata) and Brazil (Rio Grande do Norte), it was observed that people living near a petrochemical complex had around a twofold increase in acute irritant symptoms of respiratory tract diseases (14). In Korea, researchers have investigated several health effects, including respiratory and allergic symptoms and the prevalence of acute and chronic diseases such as cancer, mainly because of industrial emissions (15). Among the leading causes of death worldwide are chronic respiratory diseases in South Asia, which includes Bangladesh, Pakistan, and India. The main causes of this are increased outside air pollution from vehicle traffic and industrial pollutants, and indoor air pollution from solid fuels used for heating and cooking (16). Due to the high population density and industrial activity in Pakistani cities like Lahore, Karachi, and Islamabad, outdoor air pollution is thought to be a contributing cause of respiratory health issues (17). Due to the poor Air Quality Index (AQI), Pakistan is ranked as the 2nd most polluted country (18). In Karachi, air pollution has grown to be a major environmental problem, particularly in nearby industrial regions like Korangi, Landhi, and SITE. According to several local analyses, nitrogen dioxide (NO₂) and particulate matter (PM₂.₅) levels frequently stay beyond the WHO-recommended thresholds (19). Long-term exposure to NO₂ and PM₂ can also result in decreased lung function, chronic airway inflammation, and an increased risk of respiratory infections (20). Because they have fewer opportunity to avoid contaminated air and are exposed to pollutants for longer periods of time, populations living near industrial regions are more vulnerable, particularly children, the older adult, and people with pre-existing diseases (21). The respiratory system is known to be irritated by these pollutants, which can lead to issues including wheezing, phlegm, and persistent coughing. Similar patterns have been noted in other South Asian cities, such as Delhi and Lahore, where residents who live close to industrial areas report higher incidence of respiratory problems compared to those who live further away (22). It is estimated that about 128,000 lives are lost annually due to air pollution in Pakistan (23). Karachi is the world’s fourth-largest polluted city, experiencing high particulate matter (PM) and other pollutants (24). According to a retrospective study conducted by Hina Sharif, in a slum area of Karachi, pneumonia is one of the most common diseases, followed by bronchitis (25). Due to exceptionally high PM₂.₅ concentrations, one study carried out in Korangi and Landhi discovered that air pollution levels were higher than both Pakistan’s National Environmental Quality Standards (NEQS) and the WHO Air Quality Guidelines (22, 26). Karachi’s rapid industrialization and urbanization have led to the establishment of various industrial zones in proximity to residential areas. These industrial areas often emit pollutants into the surrounding environment, including particulate matter, gasses, and toxic substances (22). The city of Karachi is home to multiple industrial zones including, Sindh Industrial and Trading Estate, Korangi Industrial zone, Landhi Industrial Zone, Bin Qasim Industrial Park, Malir 15 Federal B Area Industrial Zone, Port Qasim Industrial Zone, North Karachi Industrial Zone, These industrial areas are known to contribute to the emissions of a number of pollutants that are harmful to human health (27). The potential health impacts of residing near industrial areas have raised concerns, particularly regarding respiratory symptoms and diseases among community residents (27). The selection of this study is based on several compelling reasons, one of the reasons is that Karachi’s industrial zones emit various pollutants that adversely affect respiratory health. Understanding the relationship between the distance from industrial areas and respiratory symptoms is crucial for assessing the health risks faced by community residents. Secondly, there is a lack of comprehensive research addressing this specific issue in Karachi, Pakistan, as only the brick kiln workers, textile workers, and cotton industry emissions were focused on previously (9, 28, 29). By engaging in this study, we aim to contribute valuable insights into the existing body of knowledge and bridge the research gap concerning the risk factors associated with residential areas near industrial zones in LMICs such as Pakistan. Our research has the potential to provide evidence-based findings that can inform policymakers, town planners, public health authorities, and community members about the health risks linked to proximity to industrial areas. Its results can facilitate the development of targeted interventions to mitigate respiratory symptoms and enhance overall wellbeing in affected communities and the findings may guide urban planning and zoning decisions to minimize residential proximity to industrial zones, promoting a healthier living environment for future generations. The main objective of the study was to determine the association between the distance from the industrial area and the frequency of respiratory symptoms [cough, wheeze, phlegm, chest tightness, and illnesses (Chronic Bronchitis and asthma)].

## 2 Literature review

Numerous studies have examined the prevalence of respiratory symptoms among industrial workers worldwide, revealing significant health impacts from exposure to industrial pollutants.

In 2020, a Korean study investigated the frequency of respiratory problems in individuals residing at varying distances from industrial regions. According to the study, people who live within 5 km away from industrial zones have a marginally higher likelihood of developing symptoms including cough (6.6% vs. 5.7%), phlegm (8.2% vs. 7.3%), dyspnea (7.9% vs. 7.1%), and wheezing (3.1% vs. 2.8%) than people who live farther away. It was found that the *p*-values for cough and phlegm were significant (15).

Residents of Parit Raja, Batu Pahat, Johor, Malaysia, who live closer to industrial regions have greater rates of respiratory problems, based on a comparative cross-sectional study done in 2024 by Khairul Nizam. The research, which comprised 110 participants in total, found that the prevalence of chronic cough was 34.5% among those who lived within 5 km of industrial areas and 9.1% among those who lived farther away. Furthermore, dyspnea was observed at a rate of 25.5% for people living within 5 km and 7.3% for people living farther away. The findings of the research showed a significant association between living close to an industrial region and developing chronic coughing and dyspnea (30).

In another cross-sectional comparison study conducted in Malaysia by Aida Farihah Mohmad Shamsuddin (31), the sample size of 104 participants. Rates of chronic cough (16.2% within 5 km vs. 5.8% more than 5 km), chronic phlegm (21.2% vs. 3.8%), chronic wheeze (5.8% vs. 7.7%), and chronic dyspnea (11.5% vs. 3.8%) were found. According to the study, people who lived within 5 km of industrial regions had much greater rates of chronic cough, chronic phlegm, and chronic wheezing than people who lived more than 5 km away. The observed differences between the two studies can be attributed to several factors, such as environmental conditions and regional differences in pollution and industrial activity levels (31).

Research conducted in Pakistan, including studies from Aga Khan University, Karachi, has demonstrated a robust association between residing near industrial areas and respiratory illnesses (32). The “Impact of Industrial Air Pollution on Human Health” study estimates that industrial pollution causes 22,000 premature adult deaths and 700 early deaths in children yearly. Respiratory symptoms were the leading cause of the estimated 60% of annual Disability Adjusted Life Years lost after mortality (33).

Khan et al. (9) found that respiratory symptoms, such as coughing, chest tightness, and shortness of breath, were highly prevalent among 800 textile workers in Faisalabad, Punjab, especially among those employed in the cotton industry’s working industry (9).

A cross-sectional study conducted in Jhang, Pakistan, by Ahmad et al. (34), with a sample size of 300, reported that 23% of industrial workers experienced chest tightness, and 18.7% suffered from chronic cough. The study also found higher rates of these symptoms among workers aged 45 years and older, those with limited formal education, non-users of masks, and individuals directly exposed to cotton dust (34).

Abbasi et al. (35): A cross-sectional survey conducted in Khairpur, Sindh, Pakistan, among 200 adults in Mehtani and Mian Jan Muhammad Abbasi villages, reported the following prevalence rates for respiratory symptoms: cough (17.5%), phlegm (19%), wheeze (14%), and shortness of breath (21%) (35).

Pakistan’s air quality has significantly declined due to industrialization. Rapid urbanization and unplanned manufacturing activities contributed to notable decreases in air quality in Karachi, one of the major cities in South Asia. Idrees et al. (22) indicated that residents living near industrial zones like SITE, Korangi, and Landhi experienced higher respiratory symptom rates than those living in less polluted areas (22).

Nafees et al. (29) observed that the following respiratory symptoms were most common in 372 adult male textile workers in Karachi, Pakistan: chronic cough (7.5%), chronic phlegm (12.9%), wheezing with shortness of breath (22.3%), and grade 2 dyspnea (21%). According to the study, respiratory complaints among textile workers in Karachi are relatively common, especially wheezing with shortness of breath and persistent phlegm (29).

In a cross-sectional survey conducted by Shaikh et al. (28) in the districts of Larkana and Dadu, Sindh, 340 adult male brick kiln workers reported the following respiratory symptoms: chronic cough (22.4%), chronic phlegm (21.2%), shortness of breath with wheezing (13.8%), chronic bronchitis (17.1%), and asthma diagnosed by a physician (8.2%) (28).

A case study on the impact of industrial pollution on human health among 150 participants residing near the Sindh Industrial Trading Estate area in Karachi by Muhammad Faisal Hayat (2023) revealed that industrial pollution hurts human health. The most prevalent health issues reported were skin diseases (48.7%), followed by diarrhea (36%), cold/cough (10%), dysentery (2.7%), headaches (2.7%), and other associated health problems (36).

In Rabbani et al. (37) linked exposure to pollutants such as SO_2_, NO_2_, CO, and particulate matter with higher prevalence rates of respiratory symptoms, indicating a clear connection between industrial pollution and respiratory health issues (37).

## 3 Research methodology

### 3.1 Study design and population

**Figure 1:**
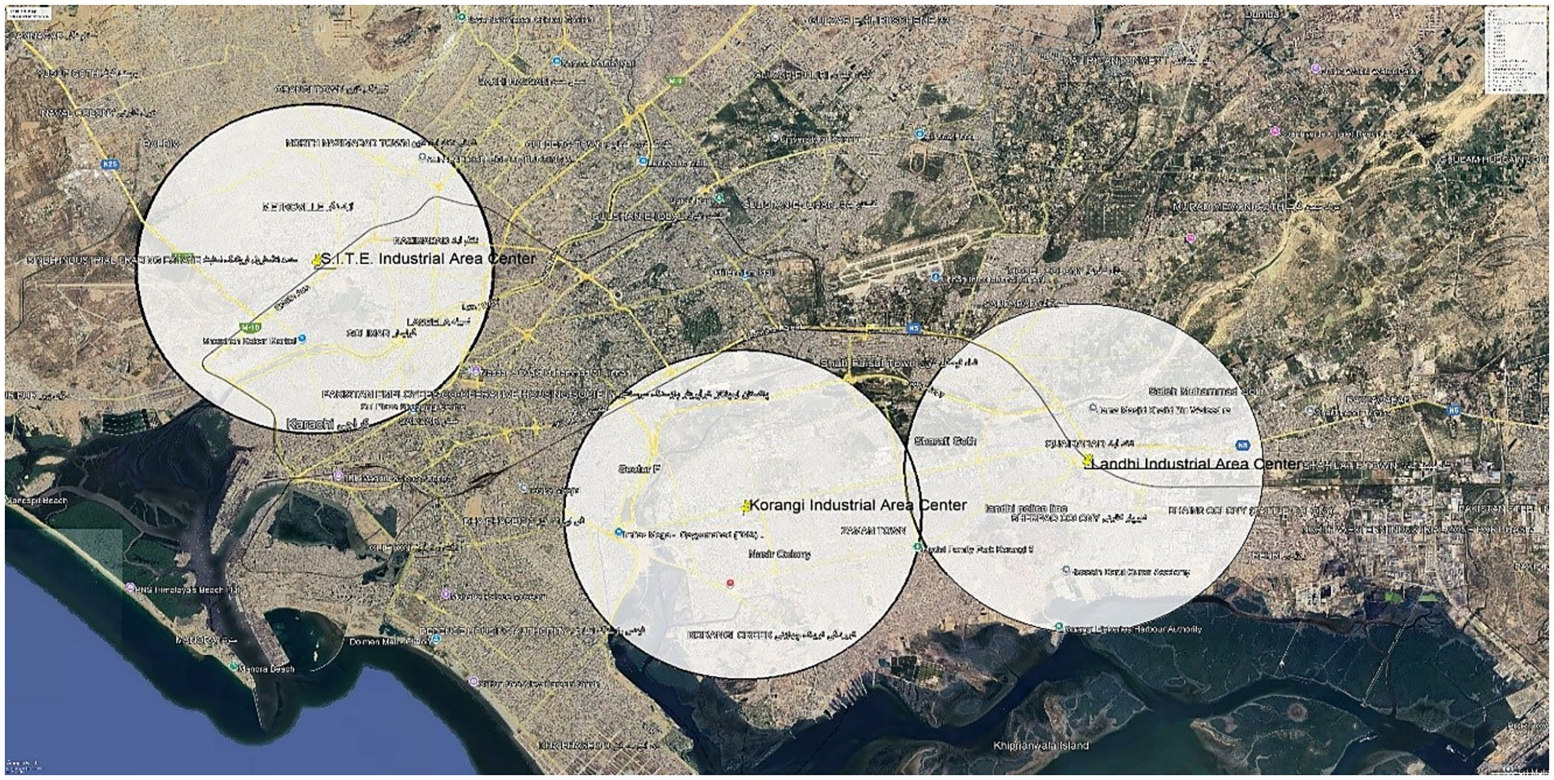
Map of the study areas showing industrial zones and their 5 km distance range in Karachi.

This was a comparative cross-sectional study from March to Aug 2024. Information on respiratory health was gathered from adults between 18 and 60 years of age living within 5 kilometers and further from industrial zones for at least 3 years. Three industrial sites were included: Korangi, Landhi, and S. I. T. E. Participants who resided within five kilometers of an industrial area were labeled as the “Proximal group,” and those who lived more than five kilometers away were considered a “Distant group.” Based on participants’ home addresses, residential proximity to industrial zones was calculated using Google Maps distance estimation. The spatial distribution of industrial areas and residential locations was illustrated in Figure 1, which was prepared using Google Earth Pro (high-resolution) for maximum visual clarity. Exclusion criteria included people who had a history of congenital respiratory conditions or Tuberculosis (TB). Participants were conveniently sampled.

### 3.2 Sample size

The sample size was calculated using OpenEpi software (Version 3.01) for comparing two proportions. We have used this formula in Equation (1).


(1) $$n=[{\left(\begin{array}{l} Z₁-\alpha /₂\surd (\mathrm{2P}(1-P))+Z₁\\ -\beta \surd (P₁(1-P₁)+P₂(1-P₂)) \end{array}\right)}^{2}]/{\left(P₁-P₂\right)}^{2}$$

Where P₁ = 31.8% (prevalence among those living within 5 km), P₂ = 20% (prevalence among those living more than 5 km away), based on a study conducted on respiratory diseases by Shiraz University of Medical Sciences, Iran (38).


$$\begin{array}{l} Z₁-\alpha /₂=1.96\;(95\%\text{confidence level}),\\ \;Z₁-\beta =0.84\;(80\%\text{power}),P=(P₁+P₂)/2. \end{array}$$

The calculated sample size was 462 participants (154 in each group).

- To ensure clarity and consistency in data collection, the following operational definitions were developed and applied for each variable included in the study.

*Human Ethics and Consent to Participate Declaration*: The study was conducted by the ethical principles outlined in the Declaration of Helsinki and approved by the Institutional Review Board (IRB) of Jinnah Sindh Medical University (Approval No: JSMU/MSPH/AIPH/021/2022). Written prior to their participation in the study, all individuals gave their informed consent.

### 3.3 Data collection and procedures

The data was gathered by three undergraduate public health students who were trained volunteers, two of whom were male and one of whom was female. Face-to-face interviews and a door-to-door strategy were used to obtain the data. Each data collector oversaw collecting information from a minimum of 154 participants in each location, 77 of whom lived within than 5 km from industrial zones and 77 of whom lived more than 5 km away.

The study tool was modified because of the initial piloting, which involved 20 participants and 5% of the population. Among these modifications were the addition of inquiries on respiratory symptoms and sociodemographic data. The tool’s final version was translated into Urdu. The Principal Investigator (PI) kept an eye on the data collectors and checked the data every day for errors or missing information. To ensure data confidentiality, the collected questionnaires were safely archived and only the co-investigators had access to them. The questionnaire was designed after a literature review.

The questionnaire used in this study to determine the frequency of respiratory symptoms was based on the validated ATS (American Thoracic Society) questionnaire. Previous research in low and middle-income countries like Pakistan has proven the validity of the ATS questionnaire (39).

The questionnaire gathered information on socio-demographic factors such as (age, gender, education, income), lifestyle factors (smoking status, mask use outdoor) occupational factors (type of work, working hours) and environmental exposure (distance from the industrial area). It also included questions on respiratory symptoms, include cough, phlegm, wheezing and respiratory illness asthma, and chronic bronchitis. Most variables were categorical, while age and years of work were continuous. Diagnoses of asthma and chronic bronchitis were based on participants’ self-reported symptoms according to the ATS questionnaire and were not clinically confirmed.

### 3.4 Statistical analysis

IBM SPSS Version 25 was used for data analysis. Descriptive statistics were first calculated for socio demographic variables as Mean and standard deviation for quantitative variables and frequencies and proportions for categorical variables. To ensure accuracy, incomplete surveys and answers with missing information were removed by list from the final analysis. Prevalence estimates determined proportions for categorical variables. Prevalence estimates determined the frequency and percentages of chronic respiratory symptoms and illnesses. The difference in the proportion of respiratory symptoms and illnesses between the two groups was determined through a chi-square test. Univariate and multivariable logistic regression analyses determined the unadjusted and adjusted relationships between each independent variable and the dependent variables (chronic bronchitis and asthma). Odds ratios (ORs) with 95% confidence intervals (CIs) were reported. *p* < 0.05 were considered significant (Table 1).

**Table 1:** Operational definitions.

Variable	Definition
Proximity group	The people who live within 5 km of territory from the industrial area (51)
Distant group	The people who live at a more than 5 km distance from the industrial area (51)
Ever smoker	(Current or former) is termed as a person who had more than 20 packs of cigarettes during life, or it may be defined as a person who had more than 1 cigarette per day for a year (29)
Former smoker	Is termed as an adult who had a minimum of a 100 cigarettes during life, but at the time of the interview, he/she had quit smoking completely (52)
Never smoked:	A person who has had less than 20 packs of cigarettes during life, or it may be termed as less than 1 cigarette per day in a year (28)
Respiratory symptoms:	The symptoms are wheezing, difficulty breathing, tightness in the chest, and cough (53)
Chronic cough	It is termed as a person who coughs nearly 4–6 times in a day, which occurs for many days in the week (more than 5 days) for a minimum of 3 months of the year and which occurs for a minimum of 2 consecutive years (28)
Chronic phlegm	It is termed as sputum expulsion nearly two times a day for many days of the week (more than 5 days) for a minimum of 3 months of the year for a minimum of two consecutive years (28)
Chronic bronchitis	Is the expectoration of cough and sputum that happens most days of the week (more than 5 days) for at least 3 months of the year for a minimum of two consecutive years (28)
Asthma	It is termed as when a minimum of two or more attacks of shortness of breath along with wheezing (whistling sound on expiration) within the past 2 months, with normal breathing in between episodes of shortness of breath, or if a physician diagnoses the person as an asthma patient (28)
Dyspnea	This term is defined by dividing into the following grades Grade 0: Shortness of breath with strenuous exercise. Grade 1: Shortness of breath when hurrying or walking up a slight hill. Grade 2: Walk slower than people of the same age because of breathlessness. Grade 3: Stops for breath after walking about 100 yards. Grade 4: Too breathless to leave the house (28)

## 4 Results

The data were entered, cleansed, and statistically examined before the results were presented. Following a summary of the participants’ sociodemographic characteristics using descriptive statistics, studies examined the association between respiratory symptoms and the proximity to industrial areas. The socio-demographic characteristics of the study participants (*n* = 462) is shown in Table 2. The participants mean age was 34.20 years (SD = 8.50 years). The gender split was equal, with males and females each representing 50% of the sample. More than one-fourth (28.8%) had no formal education. Approximately half (51.5%) had a monthly income of less than 34,000 Rs. Most participants were non-smokers (88.1%).

**Table 2:** Socio-demographic characteristics of study participants.

Variable	Categories	n %
Age (years) Mean(SD): 34.20(8.50)	18–39 40–60	**335 (72.51%)** 127(27.5%)
Sex	Male Female	231(50%) 231(50%)
Residency location	Korangi S. I. T. E Landhi	**154(33.3%)** **154(33.3%)** **154(33.3%)**
Level of education	No Education 1st to 10th education Intermediate and Above	133(28.8%) 109 (23.6%) **220 (47.6%)**
Occupation	Industrial worker Non- industrial worker	65(14.1%) **397(85.9%)**
Job experience	Less than 6 years Above 6 years	196(42.4%) **266(57.6%)**
Monthly income (Mean: 37,857 Rs: SD:19,621.98)	<37,000 Rs >37,000 Rs	238(51.5%) **224(48.5%)**
Residency duration	Less than 6 years More than 6 years	220(47.6%) **242(52.4%)**
Mask use outdoors	Yes No	171(37%) **291(62.6%)**
Smoking status	Non-Smokers Smokers	**407(88.1%)** 55(11.9%)

Bold values indicate key variables or categories highlighted for emphasis.

**Figure 2:**
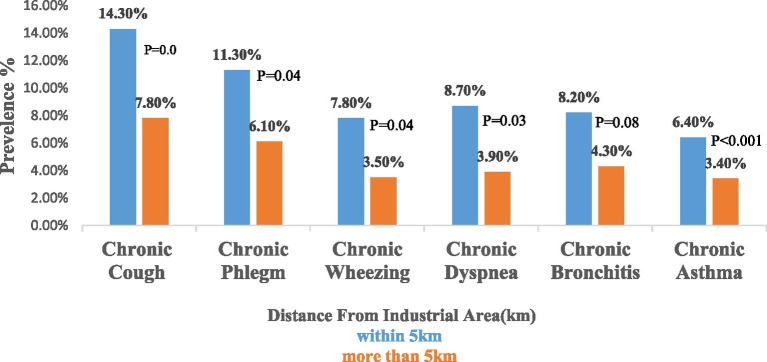
Prevelence of respiratory symptoms by distance from industrial zone.

Figure 2 indicates a significant difference in the prevalence of respiratory symptoms based on the distance from the industrial area. Individuals residing within 5 km of industrial zones exhibit higher rates of chronic cough (14.3% vs. 7.8%, *p* = 0.02), chronic phlegm (11.3% vs. 6.1%, *p* = 0.04), chronic wheezing (7.8% vs. 3.5%, *p* = 0.04), chronic dyspnea (8.70% vs. 3.9%, *p* = 0.03) chronic bronchitis (8.2% vs. 4.3%) and Chronic asthma (6.4% vs. 3.4%) compared to living more than 5 km away (*n* = 462). Although the prevalence of chronic cough was higher than that of chronic bronchitis and asthma in the study population, chronic bronchitis and asthma were selected as the primary dependent variables due to their clinical relevance, chronic nature, and greater importance in public health research. These conditions are recognized as indicators of long-term respiratory impacts associated with environmental exposures, particularly in industrial settings.

Table 3 shows the univariate and multivariable analysis of factors associated with chronic bronchitis. The likelihood of chronic bronchitis was significantly higher among individuals aged 40–60 years compared to those aged 18–39 years (AOR = 7.09, 95% CI: 2.33–21.6, *p* < 0.001). Education level showed a protective effect, as participants with intermediate or higher education had lower odds of chronic bronchitis (AOR = 0.11, 95% CI: 0.03–0.45, *p* = 0.002). Regarding environmental factors, residents living more than 5 km from industrial areas were significantly less likely to have chronic bronchitis compared to those within 5 km (AOR = 0.09, 95% CI: 0.02–0.72, *p* = 0.02). Occupational and lifestyle factors also showed important associations: participants with more than 6 years of work experience (AOR = 4.97, 95% CI: 1.34–18.4, *p* = 0.01), those with higher monthly income (AOR = 4.26, 95% CI: 1.43–12.6, *p* = 0.01), and smokers (AOR = 14.6, 95% CI: 4.06–52.6, *p* < 0.001) had significantly higher odds of chronic bronchitis. On the other hand, wearing a mask outdoors was associated with a lower likelihood of chronic bronchitis (AOR = 0.09, 95% CI: 0.012–0.72, *p* = 0.02).

**Table 3:** Univariate and multivariable analysis of the association between independent variables with chronic bronchitis.

Chronic bronchitis				
Categories	Independent variable	Unadjusted OR (95% CI)	Adjusted OR (95% CI)	*P*-value
Demographic factor	Age 18–39 40–60	References 4.16(1.92–8.98)	References 7.09(2.33–21.6)	<0.001
	Sex Female Male	References 4.14(1.65–10.3)	References 1.54(0.34–6.87)	0.56
	Education No education 1–10 Intermediate and above	References 0.99(0.39–2.50) 0.47(0.19–1.17)	References 0.44(0.12–1.65) 0.11(0.03–0.45)	0.228 0.002
Environmental factors	Residency place Korangi S. I. T. E Landhi	References 1.0(0.42–2.38) 0.61(0.23–1.64)	References 1.94(0.60–6.20) 0.38(0.10–1.39)	0.21 0.14
	Residence duration Less than 6 years More than 6 years	References 2.11(0.94–4.74)	References 1.30(0.40–4.16)	0.65
	Distance from industrial area Within 5 km More than 5 km	References 0.50(0.22–1.11)	References 0.09(0.02–0.72)	0.02
Occupational factors	Occupation Non-industrial worker Industrial worker	References 5.95(2.71–13.0)	References 2.84(0.89–8.99)	0.76
	Work experience Less than 6 years More than 6 years	References 1.68(0.75–3.79)	References 4.97(1.34–18.4)	0.01
	Monthly income <37,000 >37,000	References 1.54(0.72–3.31)	References 4.26(1.43–12.6)	0.01
Protective measures	Mask use outdoors No Yes	References 0.11(0.02–0.49)	References 0.09(0.012–0.72)	0.02
Lifestyle factors	Smoking status Non-smokers Smokers	References 10.5(4.74–23.3)	References 14.6(4.06–52.6)	<0.001

Table 4 shows the univariate and multivariable analysis of factors associated with chronic asthma. Participants with intermediate or higher education were significantly less likely to have asthma compared to those with no formal education (AOR = 0.16, 95% CI: 0.03–0.77, *p* = 0.02). Regarding environmental factors, residents of Landhi had lower odds of asthma compared to those in Korangi (AOR = 0.12, 95% CI: 0.02–0.77, *p* = 0.02). Asthma was also less common in those who lived more than 5 km from industrial regions than in people who lived within 5 km (AOR = 0.14, 95% CI: 0.03–0.67, *p* = 0.01). Additionally, occupational factors were significant: individuals with more than 6 years of work experience were more likely to have asthma (AOR = 6.86, 95% CI: 1.60–29.3, *p* = 0.01), and industrial workers had significantly higher odds of having asthma than non-industrial workers (AOR = 12.1, 95% CI: 2.95–50.0, *p* = 0.001). Smoking, mask use, and sex were among the other characteristics that did not exhibit statistically significant associations.

**Table 4:** Univariate and multivariable analysis of the association between independent variables with chronic asthma.

Chronic asthma				
Categories	Independent variable	Unadjusted OR (95% CI)	Adjusted OR (95% CI)	*P*-value
Demographic factor	Age 18–39 40–60	References 0.98(0.37–2.58)	References 0.67(0.17–2.57)	0.56
	Sex Female Male	References 3.59(1.30–9.90)	References 1.82(0.35–9.46)	0.46
	Education No education 1 to 10 Intermediate and above	References 1.07(0.37–3.05) 0.51(0.18–1.45)	References 1.14(0.26–5.01) 0.16(0.03–0.77)	0.86 0.02
Environmental factors	Residency place Korangi S. I. T. E Landhi	References 0.17(0.03–0.78) 0.80(0.32–2.00)	References 0.85(0.25–2.90) 0.12(0.02–0.77)	0.8 0.02
	Residence duration Less than 6 years More than 6 years	References 9.82(2.26–42.5)	References 3.20(0.62–16.3)	0.16
	Distance from industrial area Within 5 km More than 5 km	References 0.27(0.10–0.76)	References 0.14(0.03–0.67)	0.01
Occupational factors	Occupation Non-industrial worker Industrial worker	References 16.7(6.50–42.9)	References 12.1(2.95–50.0)	0.001
	Work experience Less than 6 years More than 6 years	References 1.61(0.64–4.03)	References 6.86(1.60–29.3)	0.01
	Monthly income <37,000 >37,000	References 0.72(0.30–1.73)	References 1.79(0.53–6.05)	0.34
Protective measures	Mask use outdoors No Yes	References 0.16(0.03–0.69)	References 0.24(0.03–1.63)	0.14
Lifestyle factors	Smoking status Non-smokers Smokers	References 11.0 (4.52–27.1)	References 2.49(0.60–10.2)	0.14

## 5 Discussion

According to this study, respiratory symptoms were significantly more common among residents who resided within 5 km from industrial regions than among those who lived more than 5 km away. According to the results, there is an apparent association between the population’s proximity to industrial regions and the risk of respiratory symptoms such chronic cough, phlegm, wheezing, and dyspnea. The observed trend underlines the impact of industrial emissions and air pollutants on respiratory health in the community and supports research showing that residing close to pollutant sources raises the risk of respiratory illnesses.

A study conducted in Korea on the adult population in 2018 found lower frequencies of respiratory symptoms in both distance categories. For example, coughing was reported by 6.6% of individuals living within 5 km of industrial areas, compared to 5.7% of those living more than 5 km away. Chronic phlegm and dyspnea showed a similar pattern with lower rates overall compared to this study (15). Another Malaysian study conducted in Parit Raja, Batu Pahat, Johor, Malaysia in 2024 also reported higher rates of respiratory symptoms among those living closer to industrial areas, with a chronic cough at 34.5% within 5 km versus 9.1% beyond 5 km, and dyspnea at 25.5% versus 7.3%. However chronic cough and dyspnea showed significant results (*p* < 0.05) in the Malaysian study (30). Although the other study population in Malaysia consisted of children aged 3–12 years, while our study focuses on adults, the findings are still noteworthy for comparison due to the similar environmental exposures related to industrial proximity. The Malaysian study, conducted in Batu Pahat, Johor in 2022 reported higher rates of respiratory symptoms among children living closer to industrial zones, including chronic cough (16.2% vs. 5.8%), chronic phlegm (21.2% vs. 3.8%), chronic wheezing (5.8% vs. 7.7%), and chronic dyspnea (11.5% vs. 3.8%). The closeness to industrial regions was significantly associated with all of these symptoms (*p* < 0.001). Although some variations in prevalence may be explained by age differences, the patterns show that exposure to industrial areas may have similar effects on respiratory health across age groups (31). The differences between this study and those conducted in Malaysia may reflect variations in the types of pollutants and levels of exposure in different industrial settings. This study found that individuals aged 40–60 years have a higher risk of chronic bronchitis which also aligns with findings from studies on brick kiln workers that show an increased prevalence of chronic bronchitis in older age group (28, 40, 41). Higher education was linked to lower risks of chronic bronchitis and asthma, likely due to better health awareness, as also supported by a study from South Africa (42). Mask use was protective, as also seen in studies from Malaysia, UAE, and China (31, 43, 44). While long-term residence near industrial areas was associated with chronic bronchitis and asthma, gender showed no significant impact, consistent with findings from studies in Ethiopia and Nepal (45, 46). The current research involved a cross-sectional, community-based study design, wherein self-administered questionnaires were used to evaluate respiratory symptoms. Community-based surveys have been the norm in similar studies in Ethiopia and Malaysia (31, 47). However, the present research refers to different methodological approaches that were used by some researchers. For example, research from Pakistan and the United Arab Emirates (UAE) on the industrial workers has integrated spirometry or peak expiratory flow rate (PEFR) testing to provide clinical confirmation of respiratory diseases, whereas others have depended on the monitoring of air quality to measure the concentrations of PM₂.₅, PM₁₀, and gaseous pollutants directly (29, 43). There are also some transitional studies in China and India that have come after the participants to record their respiratory health changes due to industrial emissions (44, 48).

Similar to the current study conducted in Karachi, several international studies have also reported consistent findings linking residential proximity to industrial areas with adverse respiratory outcomes. A population-based study in Italy revealed that people living within 5 km from the petrochemical plants had a higher prevalence of chronic cough and wheezing than those who lived at a greater distance (47). Likewise, a study from Canada reported that long-term exposure to industrial emissions was associated with reduced lung function and a higher risk of respiratory infections among nearby residents (49).

### 5.1 Strengths of the study

- The study provides a broader perspective by examining multiple industrial zones in Karachi rather than a single area, which enhances the generalizability of the findings. Similar multi-site approaches have been used in studies from Italy and Korea, improving the robustness of environmental exposure assessments (5, 47).
- The results provide convincing arguments to the decision-makers and the city planners on the necessity of creating and executing laws which would ensure the existence of an adequate safe space between housing areas and industrial plants so as to reduce the occurrence of respiratory diseases caused by the exposure of pollutants. Similar advice has also been given in research conducted in Malaysia where the significance of zoning and establishing health policies based on the distance is emphasized (30).

### 5.2 Limitations of the study

- *Measurement*: The accuracy of disease classification may have been impacted, and misclassification bias may have been introduced, by the study’s rely on self-reported respiratory symptoms and lack of objective diagnostic methods like spirometry. Similar limitations were observed in research done in India, where insufficient diagnostic resources also led to the use of self-reported results (48).
- *Sampling:* Due to practical reasons, convenience sampling was employed, which may have limited the sample’s representativeness and introduced selection bias. Comparable Malaysian studies that examined community exposure close to industrial zones also used this method (31).
- *Design:* The cross-sectional design limits the ability to draw conclusions about the causal relationship between respiratory outcomes and occupational exposure. This restriction aligns with research from Italy and Korea, where cross-sectional methods were employed to find associations rather than prove causality (15, 47).
- *Geographic scope:* Because the study was restricted to a small number of industrial zones in Karachi, the findings might not be applicable to other regions with distinctive industrial emissions, environmental conditions, or socioeconomic circumstances.
- *Responder bias:* Social desirability or subjective opinions may cause participants to over report or underreport symptoms. Similar self-reporting biases were also recognized in studies conducted in Ethiopia and Malaysia to evaluate respiratory outcomes in exposed populations (30, 45, 50).
- *Exposure duration:* Some participants had resided in industrial areas for more than 6 years, which may have affected the relationships with chronic respiratory disorders that were discovered. This limitation has also been shown in Iranian longitudinal comparisons, where longer exposure durations are linked to stronger respiratory effects (38).
- Because of this limitation, the research findings are limited to the chosen industrial zones and might not accurately represent the conditions in other industrial regions or places.

## 6 Conclusion and recommendations

In Karachi, residing within 5 km of major industrial zones is linked to a higher burden of self-reported respiratory symptoms and chronic respiratory disease. These findings underscore the importance of early screening and preventive strategies for nearby communities and support zoning and urban-planning measures that increase residential buffers from industrial facilities.

### 6.1 Policy

Enforce stricter industrial emission regulations and conduct routine air quality monitoring. Enforce the rule that residential areas and industrial zones must be at least five kilometers apart. Establish a comprehensive national strategy to reduce industrial pollution and ensure that industries adopt safer and more environmentally friendly practices.

### 6.2 Community health

People should be encouraged to use protective masks, particularly in areas with high industrial activity. Regular health screenings and examinations for industrial workers and residents near industrial areas are necessary to enable the early identification and treatment of respiratory issues. Involve and support local communities through health education and awareness programs to improve overall health outcomes.

### 6.3 Urban planning

To make sure that new industrial projects consider potential health effects on nearby populations, urban planners should incorporate health impact studies into zoning and development procedures.

### 6.4 Future research direction

Future studies should employ objective exposure and outcome evaluation methods, such as spirometer testing and air quality monitoring, to provide a more complete picture of industrial pollution and respiratory health.

## Data Availability

The original contributions presented in the study are included in the article/supplementary material, further inquiries can be directed to the corresponding author.
